# Chromophobe thyroid carcinoma: a distinct entity associated with *TSC* gene alterations

**DOI:** 10.1007/s00428-025-04380-3

**Published:** 2026-01-02

**Authors:** Agnes S. Harahap, Marc P. Pusztaszeri, Rayan Rammal, William D. Foulkes, Yuri E. Nikiforov, Jason K. Wasserman, Raja R. Seethala

**Affiliations:** 1 Department of Anatomical Pathology, Faculty of Medicine, Universitas Indonesia/Dr. Cipto Mangunkusumo Hospital, Jakarta, Indonesia; 2 Department of Pathology, McGill University, Montreal, QC Canada; 3 Department of Pathology, Memorial Sloan Kettering Cancer Center, New York, NY USA; 4 Department of Human Genetics, McGill University, Montreal, QC Canada; 5 The Lady Davis Institute for Medical Research, Jewish General Hospital, Montreal, QC Canada; 6 Cancer Research Program, Centre for Translational Biology, The Research Institute of the McGill University Health Centre, Montreal, QC Canada; 7 Department of Pathology, University of Pittsburgh Medical Center, Pittsburgh, PA USA; 8 Department of Pathology and Laboratory Medicine, The Ottawa Hospital, Ottawa, ON Canada

**Keywords:** Chromophobe, Thyroid carcinoma, Tuberous sclerosis, *TSC1*, *TSC2*

## Abstract

Chromophobe thyroid carcinoma (CTC) is a rare thyroid carcinoma with distinct morphology, often occurring in patients with tuberous sclerosis complex (TSC). The molecular drivers remain poorly understood. We report two additional CTC cases and review the literature including genetic query. Patient 1, an 11-year-old boy, had a 10.3-cm bilateral thyroid mass with tumoral capsular and vascular invasion. Microscopically, the tumor showed trabecular and nested growth, prominent cell membranes, raisinoid nuclei, eosinophilic granular cytoplasm, and perinuclear halos resembling chromophobe renal cell carcinoma. The tumor expressed thyroid markers (TTF-1, PAX-8, thyroglobulin) and renal cell markers (colloidal iron, parvalbumin, CD117) with peripheral mitochondrial accentuation by anti-mitochondrial staining. A somatic *TSC2* p.Y1650Cfs*4 frameshift mutation was identified. Patient 2, a 24-year-old woman, had a 3.8-cm infiltrative, angioinvasive right lobe tumor with similar morphology and marker expression and positive lymph nodes. Molecular analysis showed somatic *TSC2* c.2071dupC p.R691Pfs*12 and *TSC2* c.2353C > T p.Q785* mutations. A cBioPortal query (2285 cases) found *TSC1* alterations in 2% and *TSC2* in 1%, mostly deletions, enriched in poorly differentiated and anaplastic thyroid carcinomas. Literature review identified seven CTC cases; 42.8% had TSC association and 42.8% showed locoregional recurrence. Median follow-up of 36 months showed all patients alive. Common morphologic and immunophenotypic features were consistent; 85.7% exhibited vascular invasion. None harbored common mutations like *BRAF* p.V600E and *RAS.* CTC is a distinct, rare thyroid carcinoma often linked to TSC or *TSC* mutations, characterized by unique morphology and immunophenotype.

**Supplementary Information:**

The online version contains supplementary material available at 10.1007/s00428-025-04380-3.

## Introduction

As of the fifth edition of the World Health Organization classification of tumors, the most common types of thyroid cancer have been well characterized both morphologically and molecularly, including distinct types associated with familial syndromes such as cribriform morular thyroid carcinoma, which is associated with Wnt pathway alterations and familial adenomatous polyposis [1]. Despite this, novel types and subtypes continue to be described.

Chromophobe thyroid carcinoma (CTC) was first described by Hirokawa et al. [2] in 2017 as chromophobe renal cell carcinoma-like thyroid carcinoma (ChRCC-TC), a term introduced to denote a rare and distinct thyroid carcinoma that histologically resembles its namesake, chromophobe renal cell carcinoma (ChRCC). Hitherto, only five cases have been reported [2–4]. While some of these cases were associated with tuberous sclerosis complex (TSC), the molecular underpinnings of CTC are not well characterized.

TSC is a rare multisystem genetic disease caused by germline pathogenic variants in the *TSC1* (10–20%) or *TSC2* (70–90%) genes, leading to upregulation of mTOR (mammalian target of rapamycin) signaling pathway and the development of multiple tumors across various organs [5]. The most common tumors associated with TSC are benign and occur in the skin (facial angiofibroma, 57.3%), kidneys (angiomyolipoma, 47.2%), heart (cardiac rhabdomyoma, 34.3%), and brain (subependymal giant-cell astrocytoma, 24.4%) [6]. Although rare, malignant tumors have also been reported in TSC, affecting the kidneys (renal cell carcinoma, 47.7%), breast (10.8%), thyroid (9.2%), testis (7.7%), and ovary (6.2%) [7]. While thyroid malignancies have been documented in TSC, overall thyroid involvement remains relatively uncommon and is more typically reported as thyroid adenomas or nodular follicular disease [8, 9].

We herein describe the clinicopathologic and comprehensive molecular features of two additional cases of CTC harboring somatic *TSC2* driver mutations and review the clinical and genomic data in the literature on this entity and *TSC1*/2 driver alterations in thyroid carcinomas as a whole.

## Materials and methods

Two patients (one shared case between University of Pittsburgh Medical Center, Pittsburgh, USA and the Ottawa Hospital, Ottawa, Canada, and one case from McGill University, Montreal, Canada) were identified. Clinicopathological and immunohistochemical features were recorded as detailed in the results section. For both cases, next generation sequencing (NGS) was performed using a targeted ThyroSeq® v3 Assay and Oncomine™ Comprehensive Assay v3 using an Ion Torrent™ NGS platform (Thermo Fisher Scientific, Waltham, MA, USA) as previously described and according to manufacturer’s instructions [10, 11]. Additionally, an RNA whole transcriptome assay for fusions was performed.

Literature review was performed in PubMed (https://pubmed.ncbi.nlm.nih.gov) using search terms “chromophobe renal cell carcinoma-like thyroid carcinoma,” as well as “thyroid carcinoma AND tuberous sclerosis.” The prevalence and distribution of *TSC1* and *TSC2* driver alterations in thyroid carcinoma were queried in the large genomic dataset in cBioPortal (www.cbioportal.org).

The study was conducted in accordance with the principles of the Declaration of Helsinki as revised in 2013. For patient 1, ethical approval was obtained from the Institutional Review Board (IRB) of the University of Pittsburgh Human Research Protection Office, which also approved the waiver of informed consent (STUDY25070102). For patient 2, ethical approval was obtained from the IRB of the McGill University Health Centre Research Ethics Board (Project No. MP-37–2019−4865), and written informed consent for participation and publication was obtained from the patient.

## Results

### Patient 1

#### Clinical presentation

An 11-year-old boy presented with a few years history of a progressively enlarging right-sided predominant neck mass that had fluctuated in size and prominence over 1 year prior to presentation but was otherwise asymptomatic. Past medical history included cyclic neutropenia since birth, global developmental delay, and obesity. The family history was notable for an identical twin brother who also presented with a left-sided neck mass, though this had disappeared spontaneously without treatment. There was otherwise no family history of thyroid disease.

**Fig. 1:**
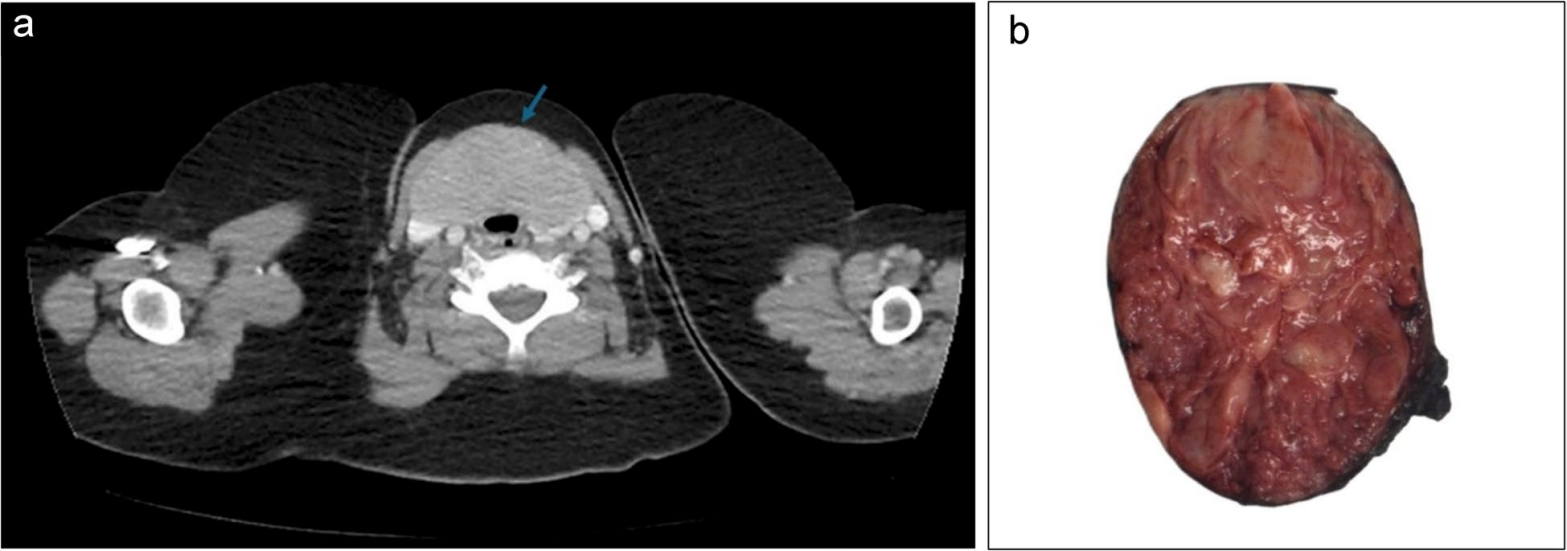
Patient 1. **a** Contrast-enhanced CT scan demonstrating diffuse thyroid involvement extending across the midline (arrow). **b** Gross photograph of the cut surface showing an expansile, well-demarcated, lobulated tan-brown mass confined to the thyroid gland

The laboratory tests revealed a marginally elevated free T3 (7.7 pmol/L), but normal TSH and free T4 levels. A contrast-enhanced computed tomography (CT) scan of the neck revealed a 10.1 × 7.7 × 4.9 cm neck mass with likely origin from the thyroid gland and extending across the midline and causing mass effect to the upper trachea and right internal jugular vein (Fig. 1a). The mass was distinct from the thymus, and multiple prominent lateral cervical lymph nodes were evident.

A fine needle aspiration biopsy (FNAB) was performed and revealed “neoplastic cells”. A follow-up percutaneous core needle biopsy demonstrated “thyroid neoplasm” that was positive for cytokeratin, TTF-1, PAX-8, and weakly positive for thyroglobulin suggesting a follicular cell phenotype; calcitonin, synaptophysin, and chromogranin were negative. Following the biopsy, an elective total thyroidectomy was performed. No gross extrathyroidal extension was noted intraoperatively.

#### Macroscopic findings

The total thyroidectomy specimen measured 10.3 × 5.5 × 4.8 cm and weighed 144 g with a smooth external surface. The thyroid was diffusely effaced by an expansile mass with relatively well-demarcated borders, confined to the thyroid, and involving both lobes and the isthmus. The cut surface was tan/brown in color and friable without grossly evident cystic changes, necrosis, or fibrosis. Residual non-neoplastic thyroid tissue was compressed and showed no significant abnormalities (Fig. 1b).

#### Microscopic findings

**Fig. 2:**
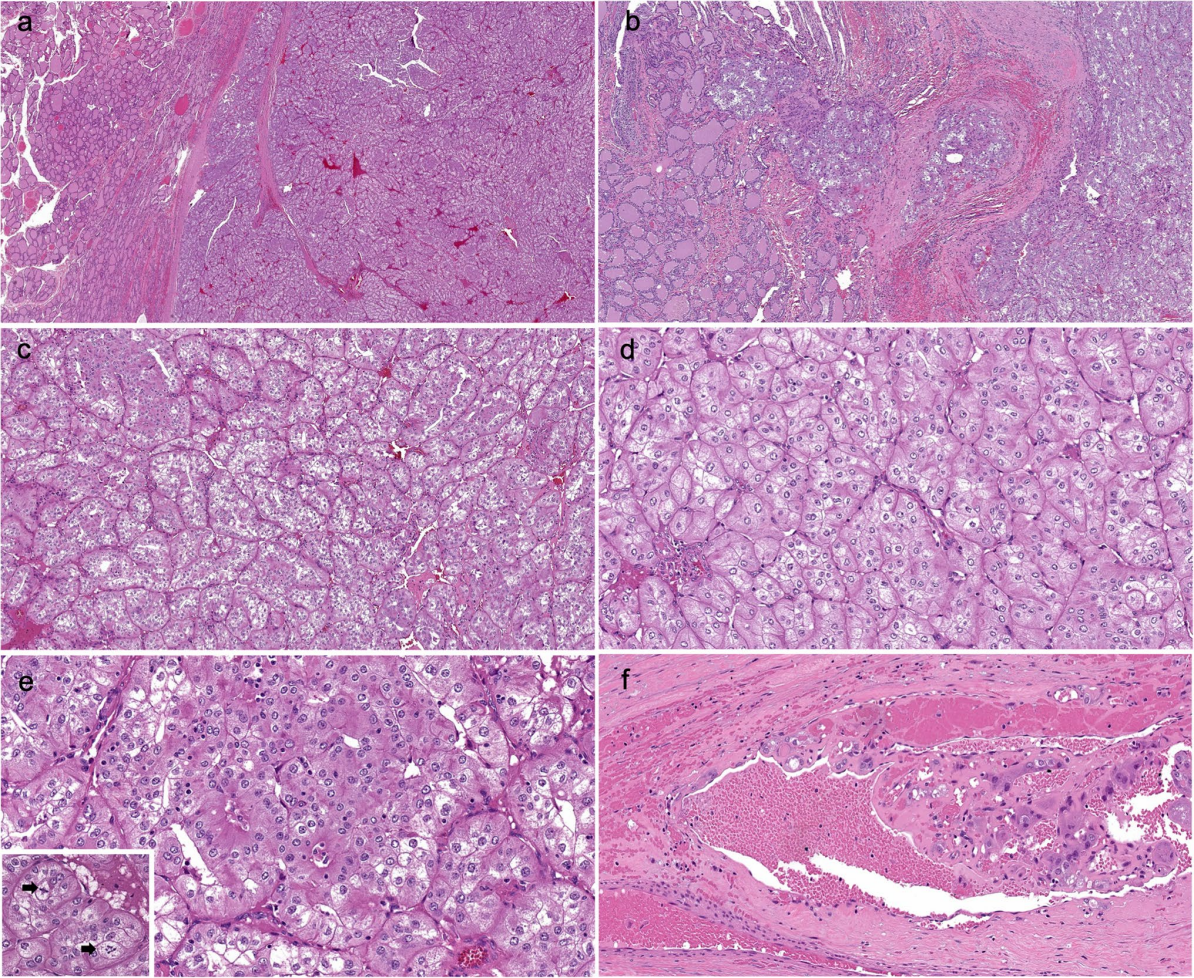
Patient 1. Histopathology. **a** The tumor is well circumscribed and confined within the thyroid gland. **b** The tumor also demonstrates capsular invasion. **c** It exhibits an alveolar and nested architecture, separated by fibrovascular septa. **d** Tumor cells are polygonal with distinct cell membranes, enlarged irregular nuclei, vesicular chromatin, and prominent nucleoli. The cytoplasm ranges from eosinophilic to clear. **e** Another area demonstrates cells with more eosinophilic, granular cytoplasm, mitosis (arrow, inset), and **f** vascular invasion

The tumor was confined to the thyroid but showed multifocal tumoral capsular invasion (Fig. 2a, b). The tumor demonstrated trabecular, alveolar, and nested growth patterns, delineated by fibrovascular septa (Fig. 2c) and consisted of large polygonal cells with prominent cell membranes, enlarged and irregular (raisinoid) nuclei, with nuclear grooves, vesicular chromatin, and prominent nucleoli, but no intranuclear pseudoinclusions. The cytoplasm varied from granular and eosinophilic to clear with distinctive perinuclear clearing or halos (Fig. 2d, e). Mitotic figures were unevenly distributed, showing a cluster of 3 in one 0.25-mm^2^ field with atypical forms, but the mitotic count was overall at a maximum of 3 per 2 mm^2^ (Fig. 2e(inset)). No tumor necrosis was observed. Additionally, calcifications resembling psammoma bodies and vascular invasion (Fig. 2f) were identified. The adjacent uninvolved thyroid was unremarkable. The tumor was initially designated as an oncocytic “chromophobe-like” subtype of papillary thyroid carcinoma.

#### Histochemistry and immunohistochemistry

**Fig. 3:**
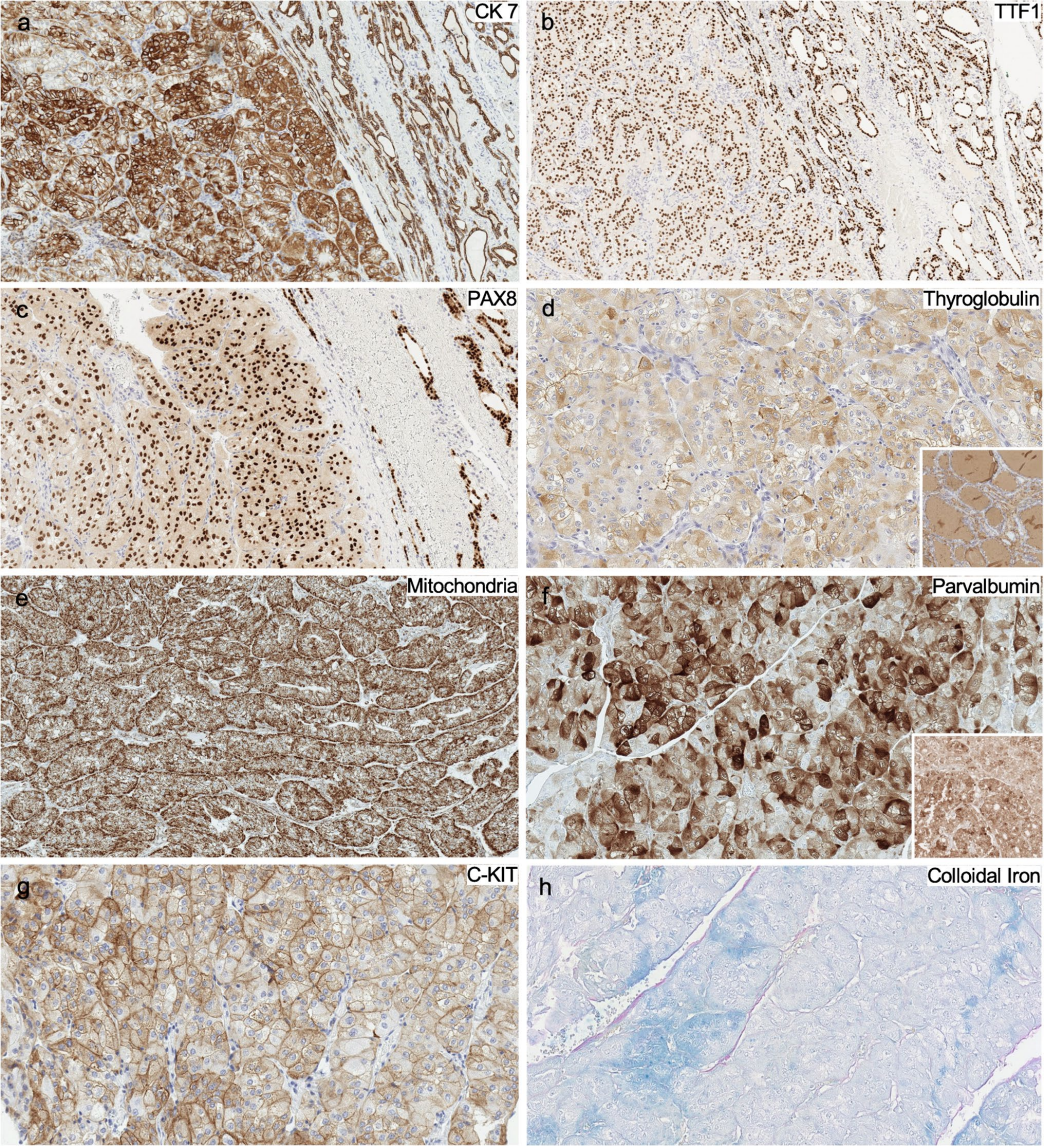
Patient 1. Immunohistochemical and histochemical staining. The tumor shows positivity for **a** CK7, **b** TTF-1, **c** PAX-8, and **d** thyroglobulin (weak). **e** Anti-mitochondrial immunostaining demonstrates peripheral accentuation. The tumor is also focally positive for **f** parvalbumin, **g** c-Kit, and **h** Hale’s colloidal iron

Ultrastructural analysis was attempted on formalin-fixed paraffin-embedded (FFPE) tissue; however, organellar structure was too poorly preserved to comment meaningfully on findings. The tumor was diffusely positive for CK7 (Fig. 3a), TTF-1 (Fig. 3b), and PAX-8 (Fig. 3c) and heterogenously positive for thyroglobulin (Fig. 3d). An anti-mitochondrial stain showed distinctive peripheral accentuation of granular staining (Fig. 3e). Parvalbumin (Fig. 3f), C-kit (Fig. 3g), and Hale’s colloidal iron (Fig. 3h) were focally positive. A Ki-67 proliferation index was low (3.6%, manual count, 1000 cells).

#### Mutational analysis

The tumor demonstrated a high level of expression of thyroid markers (*TSHR*, *KRT7*, *NIS*) by ThyroSeq, consistent with our finding on immunohistochemical analysis, and revealed a *TSC2* p.Y1650Cfs*4 frameshift mutation at 20.4% allelic frequency by Oncomine. No other alterations, including gene fusions, were detected on any of the assays including whole transcriptome analysis.

#### Follow-up

The patient showed no evidence of disease after 16 months of follow-up. Genetic consultation and germline testing revealed no clinical features of TSC and no germline pathogenic variants in the *TSC1* or *TSC2* genes.

### Patient 2

#### Clinical presentation

A 24-year-old woman, who was otherwise asymptomatic, presented with a right thyroid nodule. Past medical history included primary hyperparathyroidism with a left superior parathyroid adenoma that was removed 3 years prior. The family history was unremarkable.

A FNAB of the thyroid nodule was performed and was interpreted as Suspicious for Papillary Thyroid Carcinoma (Bethesda Category V). The material consisted of abundant follicular cell groups in a mostly microfollicular pattern, with cells displaying oncocytoid features, cytological atypia, and nuclear crowding/overlapping. These features raised the possibility of an oncocytic subtype of papillary thyroid carcinoma.

She was scheduled to have a subtotal thyroidectomy with sentinel lymph node (SLN) biopsy and central compartment dissection. However, the intraoperative exam of the SLN was positive for metastatic carcinoma resulting in conversion to a total thyroidectomy.

#### Macroscopic findings

The total thyroidectomy weighed 24.2 g. The right lobe measured 7.5 × 3.6 × 2.4 cm, and the left lobe measured 3.6 × 2.8 × 0.7 cm, both with smooth external surfaces. On serial sectioning, the right lobe contained a solid, non-encapsulated, white, rubbery nodule, measuring 3.8 × 2.9 × 2.1 cm. The remainder of the thyroid parenchyma was unremarkable.

#### Microscopic findings

**Fig. 4:**
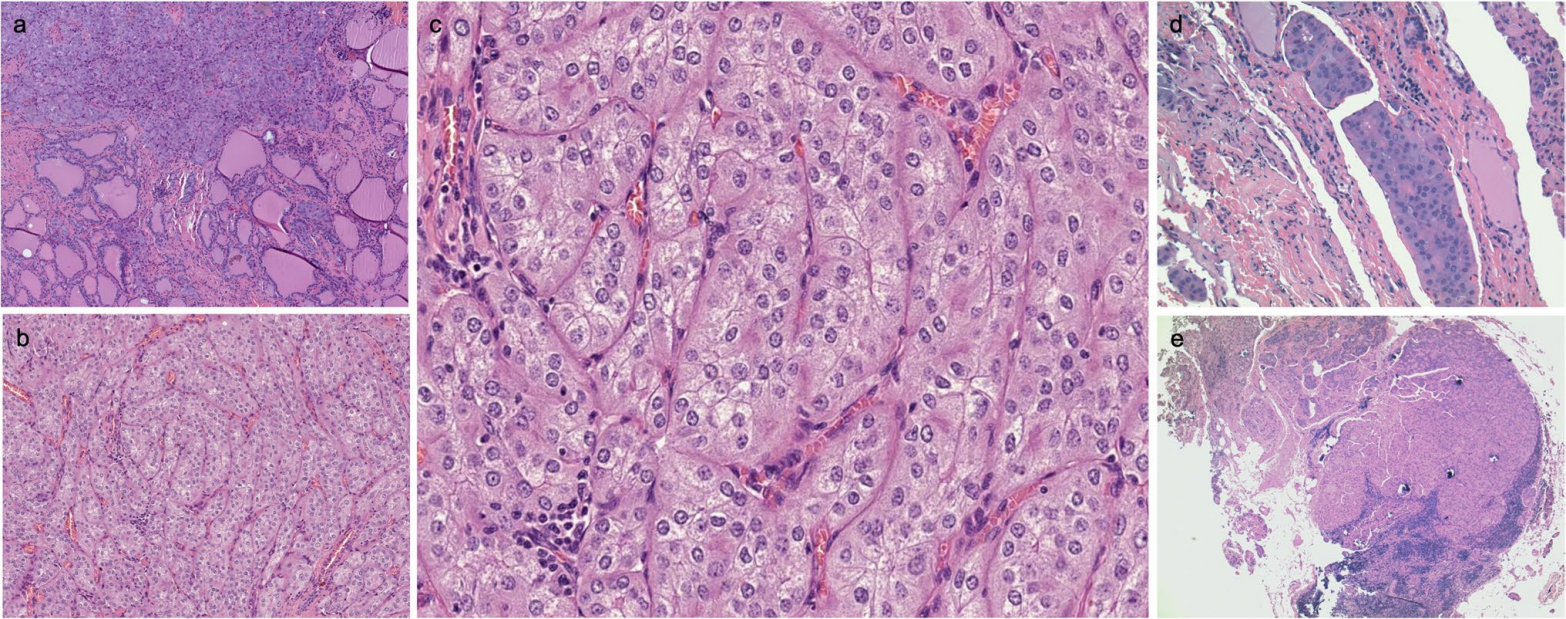
Patient 2. Histopathology. **a** Tumor-infiltrating thyroid parenchyma. **b** Solid, trabecular, and nested growth pattern lacking follicular or papillary architecture. **c** Tumor cells are large and polygonal with prominent cell membranes, enlarged irregular nuclei, vesicular chromatin, and prominent nucleoli. The cytoplasm is granular, ranging from eosinophilic to clear, with perinuclear clearing. **d** Lymphatic/vascular invasion is present. **e** Several metastases in regional lymph nodes were present. This one contains some isolated psammoma body-like calcifications

The tumor was widely infiltrative (Fig. 4a) with microscopic extrathyroidal extension into perithyroidal fibro-adipose tissue. The tumor demonstrated solid, trabecular, and insular growth patterns, delineated by fibrovascular septa, without any papillary or follicular component (Fig. 4b). It consisted of large polygonal cells with prominent cell membranes, enlarged and irregular (raisinoid) nuclei, with nuclear grooves, vesicular chromatin, and prominent nucleoli, but no intranuclear pseudoinclusion. The cytoplasm was granular and eosinophilic to clear with focal perinuclear clearing or halos (Fig. 4c). Mitotic activity was up to two mitoses per 2 mm^2^ and no tumor necrosis was observed. There was extensive lymphatic and vascular invasion (Fig. 4d). The original diagnosis was a papillary carcinoma, oncocytic subtype, with a solid/trabecular growth pattern. There were 10 out of 12 central compartment lymph nodes positive for metastatic carcinoma (up to 0.5 cm with minimal extranodal extension) (Fig. 4e).

#### Histochemistry and immunohistochemistry

**Fig. 5:**
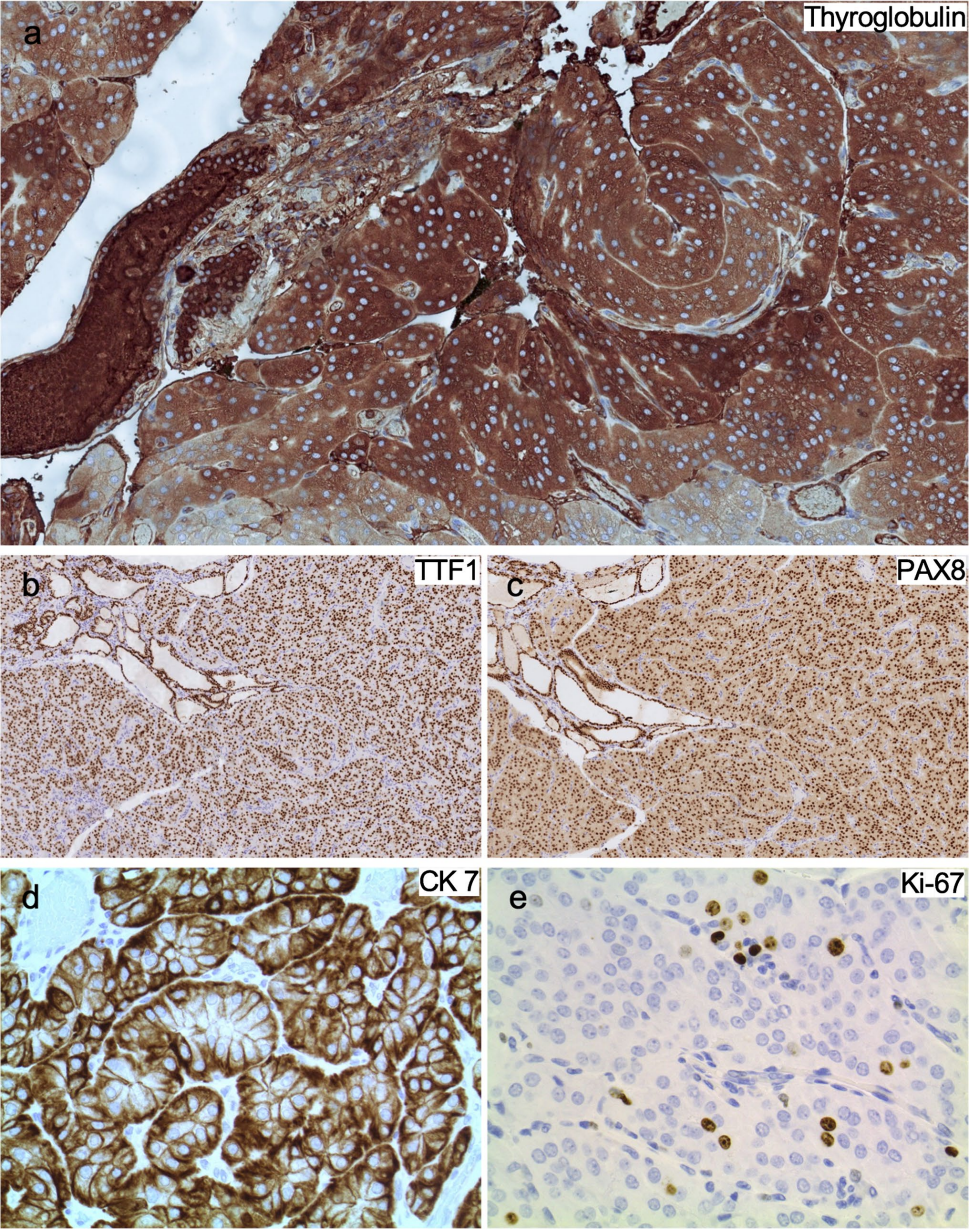
Patient 2. Immunohistochemical staining. The tumor shows variably strong positivity for **a** thyroglobulin and diffuse positivity for **b** TTF-1, **c** PAX-8, and **d** CK7. **e** The Ki-67 proliferation index is slightly increased (8–10%)

The tumor was variably strong positive for thyroglobulin (Fig. 5a) and diffusely positive for TTF-1 (Fig. 5b), PAX-8 (Fig. 5c), and CK7 (Fig. 5d). The tumor was negative for calcitonin, CD117, CA-IX, DOG-1, S-100, mammaglobin, and BRAF V600E. Hale’s colloidal iron was negative. The Ki-67 proliferation index was slightly elevated (8–10%, manual count, 1000 cells) (Fig. 5e).

#### Mutational analysis

The case demonstrated a high level of expression of thyroid markers (*TSHR, KRT7, NIS*) by ThyroSeq and showed a *TSC2* c.2071dupC p.R691Pfs*12 mutation at 36.8% allelic frequency and a *TSC2* c.2353C > T p.Q785* mutation at 27.9% allelic frequency by Oncomine, and no other alterations including fusions. Both would be expected to be disruptive to the TSC2 protein, but we do not know if the mutations are in *cis* or *trans*.

#### Follow-up

The patient received radioactive iodine (150 mCi) after surgery. She showed no evidence of disease after 4 years of follow-up. Genetic consultation did not show any clinical signs of TSC or germline pathogenic variants in either *TSC1* or *TSC2*. The patient is followed clinically and radiologically for a small ground-glass lung nodule (6 mm in the left upper lobe) and a right breast nodule compatible with fibroadenoma (1.8 × 1.8 × 1.0 cm), both found incidentally on imaging after thyroid surgery and stable in size since then.

### *TSC1 *and *TSC2* cBioPortal summary

Data from 6 datasets (2334 samples, 2285 patients) in patients with papillary thyroid carcinoma, poorly differentiated thyroid carcinoma, and anaplastic thyroid carcinoma available in cBioPortal (www.cbioportal.com) were reviewed (Supplementary Figure). Putative driver alterations were noted in 2% (40/2285) and less than 1% (19/2285) of patients for *TSC1* and *TSC2,* respectively, with a higher frequency in the 2 anaplastic/poorly differentiated carcinoma datasets with a *TSC1* alteration prevalence of 10% (28/275) and *TSC2* alteration prevalence of 7% (19/275). The majority of these were deep deletions, with only one splice mutation noted for *TSC1* (X305_splice) and one truncating mutation noted for *TSC2* (N187Qfs*2) [12–15].

## Literature review

A total of seven CTC were identified (including our cases). Four were originally reported as ChRCC-TC [2, 4]. One tumor was reported as “clear cell variant of papillary thyroid carcinoma” in the setting of TSC [3]. However, on review of the photomicrographs, the tumor resembled the other cases of CTC and is therefore included in the total count with the caveat that this is not based on a formal slide review of this case. Despite the original terminology, the current study adopts the term CTC because it more accurately reflects the tumor’s intrinsic cytonuclear morphology, avoids implying a relationship to renal neoplasia, and offers a clearer, more practical classification for diagnosis and clinical management. Other papillary thyroid carcinomas reported in the setting of TSC did not show photomicrographs for consideration [8, 16]. One reported case of medullary thyroid carcinoma in the setting of TSC remains such, based on morphologic and immunophenotypic characteristics [17].

Clinical and radiological findings for these seven CTC are summarized in Table 1 [2–4]. Median age was 19 years (range 11–60 years) with a slight male predilection of 4:3. Three (42.8%) cases had TSC by clinical and pathological criteria, with one case (case 4) showing a family history and a germline *TSC2* 4730G pathogenic variant. Radiologic findings varied and were not distinctive. For reference, thyroid disease may be noted radiologically in up to 20% of TSC patients but is typically reported as adenoma or thyroid nodular follicular disease [8, 9]. Median tumor's size was 3.8 cm (range 1.2–10.3 cm) and no case showed extrathyroidal structural involvement. Four of seven cases had nodal metastases on presentation, none with distant metastasis. Three of seven cases (42.8%) had locoregional recurrences at 6 months (case 4) and 2 years (cases 1 and 2). No cases had pathologically proven distant recurrence, though case 4 had lung nodules on imaging. Median follow-up was 36 months (range 15–156 months) with all patients alive at the end of follow-up. As seen in Table 2 [2–4], all cases had FNAB and were recognized as neoplastic, with the majority (6/7, 85.7%) designated as malignant or suspicious for malignancy. Grossly, tumors were lobulated and ranged from white to tan/brown. Initial diagnoses for all cases were malignant, with 4/7 (57.1%) considered a subtype of papillary thyroid carcinoma, and one each designated as widely invasive follicular thyroid carcinoma, poorly differentiated thyroid carcinoma, and metastatic renal cell carcinoma. Four of seven (57.1%) were encapsulated with tumoral capsular invasion, while the remainder were infiltrative, and two cases (28.6%) showed microscopic extrathyroidal extension. Six of seven (85.7%) showed vascular invasion, but only two cases had necrosis. Mitotic counts were only reported in three cases, ranging from none to 3 per 2 mm^2^. In keeping with their namesake, all CTC showed raisinoid nuclei and clear to eosinophilic cytoplasm with a perinuclear halo; only one case showed nuclear pseudoinclusions. Psammoma-like luminal calcifications were noted in 4/7 (57.1%) cases.

**Table 1:** Summary of clinical data from the present case series and from the existing literature

**Case**	**Study (Author, Year)**	**Country**	**Age (Years)**	**Sex**	**Ethnicity**	**Clinical Presentation**	**TSC-associated** **manifestations**	**Family History**	**Radiological Findings**
1	Hirokawa et al., 2017 (Case 1)^2^	Japan	15	F	Asian	Epilepsy, cardiac tumor, bilateral renal angiomyolipoma, multiple facial sebaceous adenoma, ventricular brain tumor, opthalmic hamartoma	Yes (germline mutation testing was not performed)	N/A	N/A
2	Hirokawa et al., 2017 (Case 2)^2^	Japan	19	M	Asian	N/A	No	N/A	Hot area nodule (gallium scintigraphy)
3	Hirokawa et al., 2017 (Case 3)^2^	Japan	21	M	Asian	Epilepsy, bilateral renal cysts, and renal angiomyolipoma	Yes (germline mutation testing was not performed)	N/A	A nodule measuring 16.9 mm in the right lobe thyroid (USG)
4	Flader et al., 2017^3^	Poland	13	M	Caucasian	Facial angiofibromas, hepatic and renal angiomyolipomas, left eye hamartoma, depigmented skin patches, subependymal nodule, and cortical tubers	Yes (4730G deletion, exon 36, *TSC2* gene)	The father and 2 younger sisters of the patient have TSC	Nodular left thyroid lobe, multiple hypoechoic areas with numerous microcalcifications, and increased blood flow (Doppler and CT Scan); Left lobe "cold area" (Thyroid scintigraphy).
5	Kwon and Jang, 2021^4^	Korea	60	F	Asian	No abnormalities on physical examination nor laboratory evaluation	No	Unremarkable	A 1.5 cm low echoic oval mass in the left thyroid (ultrasonography); No suspicious primary lesion in either kidney (CT scan); No other primary sites (torso PET Scan)
6	Current Study (Patient 1)	Canada	11	M	Caucasian	A progressively enlarging right neck, developmental delay, and obesity	No (germline mutation testing negative)	Identical twin brother with history of a thyroid lump	A neck mass extending across the midline and causing mass effect to the upper trachea and right internal jugular vein (CT Scan)
7	Current Study (Patient 2)	Canada	24	F	Caucasian	Right thyroid nodule, otherwise asymptomatic	No (germline mutation testing negative)	Unremarkable	N/A
**Case**	**Laterality**	**Size (cm)**	**Tumor border**	**Structures Involved**	**Treatment**	**Lymph node involvement**	**Locoregional Recurrence**	**Distant Metastasis**	**Follow Up**
1	Left	4.7	Well-demarcated	None	Left lobectomy (initial treatment) Completion total thyroidectomy with left modified neck lymph node resection (two-years after)	No	Local recurrence (2 years)	No	Alive, 13 years
2	Left	10	Infiltrative	None	Total thyroidectomy with modified left lymph node dissection (initial treatment), bilateral modified neck lymph node resections (two and four years after), and radioactive iodine therapy	Yes	Two times of regional recurrence (2 and 4 years)	No	Alive, 6 years
3	Right	1.3	Well-demarcated	None	Right lobectomy with central neck lymph node resection	Yes	No	No	Alive, 15 months
4	Bilateral/ midline	1.2	Infiltrative	N/A	Total thyroidectomy with lymph node dissection,131-I treatment	Yes	Regional recurrence (6 months)	No*	Alive, 26 months
5	Left	1.5	Well-demarcated	None	Total thyroidectomy and central lymph node dissection	No	No	No	Alive, 36 months
6	Bilateral/ midline	10.3	Well-demarcated	None	Total thyroidectomy	No	No	No	Alive, 16 months
7	Right	3.8	Infiltrative	None	Total thyroidectomy with lymph node dissection, 131-I treatment	Yes	No	No	Alive, 4 years

*F *Female, *M*-male, *N/A *not available, *TSC *tuberous sclerosis complex, *USG *ultrasonography, *CT *computerized tomography, *PET *positive emission tomography

*Unproven lung nodule

**Table 2:** Summary of pathological data from the present case series and from the existing literature

Case	Study (author, year)	FNA	Gross findings	Initial pathology diagnosis	Encapsulated	Capsular invasion	ETE^*^	Vascular invasion	Psammoma-like calcifications	Necrosis	Mitosis	Nuclear contours	Intranuclear pseudoinclusions	Cytoplasm
1	Hirokawa et al., 2017 (case 1)^2^	Malignancy, NOS	Solid, lobulated, whitish tan, partly encapsulated	FTC, widely invasive	Yes	Yes	No	Yes	Yes	Yes	N/A	Raisinoid	N/A	Clear to eosinophilic with perinuclear halo
2	Hirokawa et al., 2017 (case 2)^2^	PDTC	Solid, lobulated, whitish tan, infiltrative	PDTC	No	N/A	Yes	Yes	No	Yes	N/A	Raisinoid	N/A	Clear to eosinophilic with perinuclear halo
3	Hirokawa et al., 2017 (case 3)^2^	Oxyphiic variant PTC	Solid, lobulated, whitish tan, partly encapsulated	PTC, oxyphillic	Yes	Yes	No	Yes	No	No	N/A	Raisinoid	N/A	Clear to eosinophilic with perinuclear halo
4	Flader et al., 2017^3^	Suspicious for PTC (TBSRTC category V)	N/A	PTC, clear cell	No	N/A	No	Yes	Yes	N/A	N/A	Raisinoid	Yes	Clear to eosinophilic with perinuclear halo
5	Kwon and Jang, 2021^4^	Malignant, possibly PTC	Two distinct masses, grayish colored with a thin fibrous capsule, and homogenous cut surface	Metastatic RCC	Yes	Yes	No	No	N/A	No	None	Raisinoid	N/A	Clear to eosinophilic with perinuclear halo
6	Current study (patient 1)	Neoplastic cells	Well-demarcated, lobulated, homogenous, and tan-brown	PTC, oncocytic "chromophobe-like"	Yes	Yes	No	Yes	Yes	No	3 per 2mm^2^	Raisinoid	No	Clear to eosinophilic with perinuclear halo
7	Current study (patient 2)	Suspicious for PTC (TBSRTC category V)	Solid, non-encapsulated white rubbery	PTC, oncocytic	No	N/A	Yes	Yes	Yes	No	2 per 2mm^2^	Raisinoid	No	Clear to eosinophilic with perinuclear halo

*FNA* fine needle aspiration, *NOS* not otherwise specified, *PDTC* poorly differentiated thyroid carcinoma, *PTC* papillary thyroid carcinoma, *TBSRTC* The Bethesda System for Reporting Thyroid Cytopathology, *FTC* follicular thyroid carcinoma, *RCC* renal cell carcinoma, *N/A* not available, ETE* extrathyroidal extension (microscopically)

By immunohistochemistry (Table 3) [2–4], markers of epithelial and thyroid differentiation were consistently positive: all cases tested were CK7, TTF-1, and PAX-8 positive, with variable positivity for thyroglobulin staining. In contrast, ChRCC-type markers were more variable. The most consistent staining was the anti-mitochondrial antibody that showed the typical peripheral distribution of staining, sparing the perinuclear halo in all four cases tested. However, only 2/5 (40%) cases stained positive for Hale’s colloidal iron, 1/5 (20%) stained positive for c-Kit, and 1/3 (33.3%) showed CA-IX positivity. Only case 6 did show parvalbumin staining. Regarding immunohistochemical biomarkers, p53 showed a wild-type pattern of staining in all tested cases. The median Ki-67 proliferation index was 8.2% (range 3.5–10.5%) in the six cases tested.

**Table 3:** Summary of histochemistry, immunohistochemistry, and molecular data from the present case series and from the existing literature

Case	Study (author, year)	Histochemistry and immunohistochemistry											Molecular Study				
		Colloidal iron	CK7	Thyroglobulin	TTF-1	PAX-8	Mitochondrial	CA-IX	Parvalbumin	c-Kit	P53	Ki-67	***TSC***	***BRAF*** V600E	***NRAS***	***HRAS***	***KRAS***
1	Hirokawa et al., 2017 (case 1)^2^	Negative	Positive, weak, focal	Positive, weak, focal	Positive, moderate, diffuse	Positive, strong, diffuse	Positive, moderate, diffuse peripheral accentuation	N/A	N/A	Negative	WT	10.5%	N/A	Negative	Negative	Negative	Negative
2	Hirokawa et al., 2017 (case 2)^2^	Positive, focal	Positive, moderate, diffuse	Positive, weak, focal	Positive, moderate, diffuse	Positive, strong, diffuse	Positive, moderate, diffuse peripheral accentuation	N/A	N/A	Negative	WT	8.2%	N/A	Negative	Negative	Negative	Negative
3	Hirokawa et al., 2017 (case 3)^2^	Negative	Positive, strong, diffuse	Positive, weak, focal	Positive, moderate, diffuse	Positive, strong, diffuse	Positive, moderate, diffuse peripheral accentuation	N/A	N/A	Negative	WT	3.5%	N/A	Negative	Negative	Negative	Negative
4	Flader et al., 2017^3^	N/A	N/A	Positive, focal	Positive	N/A	N/A	N/A	N/A	N/A	N/A	Mostly negative	*TSC2* c.4730del in exon 36	N/A	N/A	N/A	N/A
5	Kwon and Jang, 2021^4^	N/A	Positive, strong, diffuse	Positive, focal	Positive, focal	Positive, strong, diffuse	N/A	Positive, strong, diffuse	N/A	N/A	WT	N/A	N/A	Negative	Negative*	Negative	Negative
6	Current study (patient 1)	Positive, weak, focal	Positive, strong, diffuse	Positive, weak, diffuse	Positive, strong, diffuse	Positive, strong, diffuse	Positive, moderate, diffuse peripheral accentuation	Negative	Positive, moderate, focal	Positive, moderate focal	N/A	3.6%	*TSC2* p.Y1650Cfs*4	Negative	Negative	Negative	Negative
7	Current study (patient 2)	Negative	Positive, strong, diffuse	Positive, variably strong/weak, diffuse	Positive, strong, diffuse	Positive, strong, diffuse	N/A	Negative	N/A	Negative	N/A	8–10%	*TSC2* c.2071dupC p.R691Pfs*12; *TSC2* c.2353C > T p.Q785*	Negative	Negative	Negative	Negative

*N/A* not available, *WT* wild type

^*^Case 5 showed a concurrent papillary thyroid microcarcinoma with an *NRAS* p.Q61R mutation

Molecular testing methodologies and gene coverage varied across studies. In our series, cases 6 and 7 demonstrated somatic *TSC2* mutations, while germline testing was negative. In contrast, three previously reported cases had clinical TSC, including one with a germline pathogenic *TSC2* variant, although their tumors were not specifically evaluated for *TSC1* or *TSC2* mutations. Notably, unlike most thyroid tumors that typically exhibit identifiable driver mutations by current genetic testing methods, such as *BRAF*-like or *RAS*-like alterations [14, 18], none of the six tumors tested showed *BRAF* p.V600E or *RAS* mutations. Case 5, however, did show a concurrent papillary thyroid microcarcinoma with an *NRAS* p.Q61R mutation.

## Discussion

CTC is exceptionally rare (estimated prevalence at a single institution of 2/12064 (0.02%)) [2], with distinctive morphologic and immunophenotypic characteristics. Patients tend to be young, and almost half the cases are known to be associated with TSC. We confirm that even seemingly sporadic cases show *TSC2* mutations. *TSC1* and *TSC2* are tumor suppressors that encode hamartin and tuberin proteins, which bind with TBC1D7 protein to form the TSC protein complex and function to maintain the inactive state of mTOR, a member of the PI3K/AKT/mTOR pathway that is vital to regulating cell metabolism, proliferation, growth, and survival [5]. Tumorigenesis is attributable to loss of function through mutation or deletion, which results in persistent mTOR activity, leading to uncontrolled cellular proliferation and formation of tumors [19, 20].

*TSC1* and *TSC2* molecular alterations are rare in thyroid cancer and usually consist of deletions in aggressive tumors (often poorly differentiated or anaplastic thyroid carcinomas). Our cBioPortal query verifies this, showing that a small subset of carcinomas (mainly anaplastic type) harbor homozygous deletions of *TSC1* (~ 1.8%) and/or *TSC2* (~ 0.8%), whereas intragenic alterations, such as single nucleotide alterations that are putatively pathogenic, as well as small indels, are much rarer. As expected, these alterations are somewhat enriched in the anaplastic and poorly differentiated thyroid carcinoma datasets. CTC conforms to the expectations of aggressive behavior with frequent adverse histologic features and locoregional spread. While no patient died of disease, follow-up is limited in most of the reported cases.

CTC thus joins the tumor types for which mTOR inhibitors such as everolimus may have value. In 2009, the U.S. Food and Drug Administration approved temsirolimus and everolimus for advanced renal cancer [21]. Since then, mTOR inhibitors have proven effective and safe in treating various TSC-related tumors, including subependymal giant-cell astrocytoma, renal angiomyolipoma, and sporadic lymphangioleiomyomatosis [22]. Relevant to thyroid, Wagle et al. reported an advanced anaplastic thyroid carcinoma patient with a *TSC2* Q1178^⋆^ truncating mutation for whom everolimus treatment resulted in an 18-month remission [23]. While further research with more patients is needed to further recommend how this entity should be managed, current data show that most patients underwent total thyroidectomy with lymph node dissection and some of the patients also received additional radioactive iodine therapy.

CTC has taxonomic implications as well. While most tumors are parsed into papillary, follicular, oncocytic (Hürthle cell), and medullary thyroid carcinoma, with high grade and anaplastic carcinomas representing progression of the follicular cell-derived lineage [1], CTC appears to be one of the few entities that does not fit into this standard rubric. An argument can be made that the nuclear features warrant classification as a papillary thyroid carcinoma subtype, and this is the most frequent initial diagnosis in the reported cases [2, 3]. However, the nuclear membrane irregularities are more exaggerated (i.e., raisinoid) without as much chromatin clearing as most papillary thyroid carcinomas, and nuclear pseudoinclusions are rare [3]. Furthermore, the nested appearance and perinuclear clearing are unique. On the other hand, a case can be made that this mitochondria-rich tumor could be classified as an oncocytic carcinoma type, but the demographics (younger age, TSC association), *TSC2* alterations, and unique morphologic features may warrant separation.

The distinct appearance and the association with TSC raise unique differential diagnostic considerations. Foremost is metastasis from a primary ChRCC, as its namesake implies. In fact, one previously reported case was initially diagnosed as such [4]. Renal involvement is frequent in TSC (80–85%) and is the most common cause of mortality in TSC patients. It includes renal cell carcinoma as well as angiomyolipoma and renal cysts [19, 24, 25]. Interestingly, while the morphologic resemblance of CTC is to ChRCC, classic ChRCC is not typically associated with *TSC* gene alterations or with TSC. However, the comparisons remain relevant, as the most frequently reported subtype of renal cell carcinoma in patients with TSC, comprising 59% of cases, showed morphologic features similar to ChRCC [26]. Other histologic subtypes, including low-grade oncocytic tumor [27], eosinophilic vacuolated tumor [28], eosinophilic solid [26], and cystic renal cell carcinoma [29] may also occur in the setting of TSC or in sporadic renal tumors harboring alterations in *TSC1*, *TSC2*, or other components of the mTOR signaling pathway. Nonetheless, while both CTC and renal carcinomas share PAX-8 expression; TTF-1 and thyroglobulin are restricted to the former, effectively resolving the differential diagnosis regardless of renal cell carcinoma type or subtype.

Distinguishing between other primary thyroid tumors is more an issue of taxonomy rather than a true diagnostic dilemma. However, medullary thyroid carcinoma, the great mimic, may show clear cell and oncocytic features and may theoretically pose a challenge. In contrast to the homogenous appearance noted in CTC, medullary thyroid carcinoma often shows a mixture of patterns, and lesional cells tend to have less cytoplasm and more cell dyshesion. When present, amyloid may be discriminatory, and immunohistochemical expression of calcitonin would resolve this differential diagnosis.

In summary, CTC is a rare and unique primary follicular-derived thyroid tumor that morphologically resembles ChRCC and occurs in younger individuals. This tumor is associated with *TSC* gene alterations. While CTC can be a manifestation of inherited TSC, it may also arise in patients without a family history of TSC, either de novo through germline pathogenic variants or as somatic *TSC1/TSC2* mutations. Recognition of CTC should prompt consideration of genetic counseling. If a germline *TSC1* or *TSC2* pathogenic variant is identified, patients should undergo comprehensive screening for other TSC-associated manifestations, including brain MRI, renal imaging, cardiac evaluation, dermatologic examination, and pulmonary assessment, in line with current clinical guidelines.

While CTC is a histologically aggressive tumor, lethality has not been documented to date. The documentation of *TSC* alterations raises the potential to target ChRCC with mTOR inhibitors. While its placement in thyroid tumor phylogeny is not yet established, we would advocate provisionally for CTC as a distinct thyroid cancer type with characteristic morphologic and molecular features.

## Supplementary Information

Below is the link to the electronic supplementary material.

**Supplementary Fig 1.** **a)** A cBioportal query of *TSC1 *and *TSC2 *driver alterations in thyroid carcinoma. The truncated oncoprint consists of data from 2285 patients (2334 samples) across 6 studies and shows 40/2285 (1.8%) *TSC1* alterations, and 19/2285 (0.8%) *TSC2* alterations. The majority for both consists of deep deletions (blue) with only one *TSC1 *splice mutation (orange dot), and one *TSC2* truncating mutation (black dot). **b)** Kaplan-Meier plots of overall survival for *TSC1 *altered, and **c)** *TSC2* altered thyroid carcinomas as compared to unaltered counterparts. Both gene alterations show worse overall survival (log rank *P* <0.001). The *TSC1 *altered hazard ratio as compared to the unaltered reference group was 9.916 (95% confidence interval: 2.98–32.994.98.994). The *TSC2 *altered hazard ratio as compared to the unaltered reference group was 18.318 (95% confidence interval: 2.888–116.186.888.186)(4.13 MB)

High Resolution Image (17.5 MB)

## Data Availability

The datasets generated during and/or analyzed during the current study are available from the corresponding author on reasonable request.

## References

[1] Jung CK, Bychkov A, Kakudo K (2022) Update from the 2022 World Health Organization classification of thyroid tumors: a standardized diagnostic approach. Endocrinol Metab (Seoul) 37:703–718. 10.3803/EnM.2022.1553 36193717 10.3803/EnM.2022.1553 PMC9633223

[2] Hirokawa M, Miyauchi A, Kihara M et al (2017) Chromophobe renal cell carcinoma-like thyroid carcinoma: a novel clinicopathologic entity possibly associated with tuberous sclerosis complex. Endocr J 64:843–850. 10.1507/endocrj.EJ17-0096 28680002 10.1507/endocrj.EJ17-0096

[3] Flader M, Kurzawa P, Maldyk J et al (2017) Papillary thyroid carcinoma in a boy with familial tuberous sclerosis complex attributable to a TSC2 deletion-a case report. Curr Oncol 24:e423–e428. 10.3747/co.24.3555 29089812 10.3747/co.24.3555 PMC5659166

[4] Kwon HJ, Jang MH (2021) Chromophobe renal cell carcinoma-like thyroid carcinoma: possible misdiagnosis as metastatic renal cell carcinoma. Int J Clin Exp Pathol 14:1095–1101 34900078 PMC8661068

[5] Uysal SP, Şahin M (2020) Tuberous sclerosis: a review of the past, present, and future. Turk J Med Sci 50:1665–1676. 10.3906/sag-2002-133 32222129 10.3906/sag-2002-133 PMC7672342

[6] Kingswood JC, d’Augères GB, Belousova E et al (2017) TuberOus SClerosis registry to increase disease Awareness (TOSCA) - baseline data on 2093 patients. Orphanet J Rare Dis 12:2. 10.1186/s13023-016-0553-5 28057044 10.1186/s13023-016-0553-5 PMC5217262

[7] Sauter M, Belousova E, Benedik MP et al (2021) Rare manifestations and malignancies in tuberous sclerosis complex: findings from the TuberOus SClerosis registry to increAse disease awareness (TOSCA). Orphanet J Rare Dis 16:301. 10.1186/s13023-021-01917-y 34229737 10.1186/s13023-021-01917-y PMC8259106

[8] Auladell M, Boronat S, Barber I, Thiele EA (2015) Thyroid nodules on chest CT of patients with tuberous sclerosis complex. Am J Med Genet A 167A:2992–2997. 10.1002/ajmg.a.37339 26332136 10.1002/ajmg.a.37339

[9] Wataya-Kaneda M, Tanaka M, Hamasaki T, Katayama I (2013) Trends in the prevalence of tuberous sclerosis complex manifestations: an epidemiological study of 166 Japanese patients. PLoS One. 10.1371/journal.pone.0063910 23691114 10.1371/journal.pone.0063910 PMC3656843

[10] Nikiforova MN, Mercurio S, Wald AI et al (2018) Analytical performance of the ThyroSeq v3 genomic classifier for cancer diagnosis in thyroid nodules. Cancer 124:1682–1690. 10.1002/cncr.31245 29345728 10.1002/cncr.31245 PMC5891361

[11] Patel S, Wald AI, Bastaki JM et al (2023) NKX3.1 expression and molecular characterization of secretory myoepithelial carcinoma (SMCA): advancing the case for a salivary mucous acinar phenotype. Head Neck Pathol 17:467–478. 10.1007/s12105-023-01524-2 36746884 10.1007/s12105-023-01524-2 PMC10293155

[12] Gao J, Aksoy BA, Dogrusoz U et al (2013) Integrative analysis of complex cancer genomics and clinical profiles using the cBioPortal. Sci Signal. 10.1126/scisignal.2004088 23550210 10.1126/scisignal.2004088 PMC4160307

[13] Landa I, Ibrahimpasic T, Boucai L et al (2016) Genomic and transcriptomic hallmarks of poorly differentiated and anaplastic thyroid cancers. J Clin Invest 126:1052–1066. 10.1172/JCI85271 26878173 10.1172/JCI85271 PMC4767360

[14] Cancer Genome Atlas Research Network (2014) Integrated genomic characterization of papillary thyroid carcinoma. Cell 159:676–690. 10.1016/j.cell.2014.09.050 25417114 10.1016/j.cell.2014.09.050 PMC4243044

[15] Zeng PYF, Prokopec SD, Lai SY et al (2024) The genomic and evolutionary landscapes of anaplastic thyroid carcinoma. Cell Rep. 10.1016/j.celrep.2024.113826 38412093 10.1016/j.celrep.2024.113826 PMC11077417

[16] Wolff M (1973) Lymphangiomyoma: clinicopathologic study and ultrastructural confirmation of its histogenesis. Cancer 31:988–1007. 10.1002/1097-0142(197304)31:4<25253C988::aid-cncr2820310433>25253E3.0.co;2-x 4706060 10.1002/1097-0142(197304)31:4<988::aid-cncr2820310433>3.0.co;2-x

[17] Dicorato P, Calvanese A, Maiuolo A et al (2009) Medullary thyroid carcinoma and tuberous sclerosis. Endocr Pathol 20:141–144. 10.1007/s12022-009-9077-z 19424876 10.1007/s12022-009-9077-z

[18] Dettmer MS (2025) The actual and future role of molecular tests in thyroid pathology. Virchows Arch. 10.1007/s00428-025-04334-9 41242985 10.1007/s00428-025-04334-9 PMC12876085

[19] Nair N, Chakraborty R, Mahajan Z et al (2020) Renal manifestations of tuberous sclerosis complex. J Kidney Cancer 7:5–19. 10.15586/jkcvhl.2020.131 10.15586/jkcvhl.2020.131 PMC7478169 32953421

[20] Henske EP, Cornejo KM, Wu C-L (2021) Renal cell carcinoma in tuberous sclerosis complex. Genes (Basel) 12:1585–1592. 10.3390/genes12101585 34680979 10.3390/genes12101585 PMC8535193

[21] Yuan R, Kay A, Berg WJ, Lebwohl D (2009) Targeting tumorigenesis: development and use of mTOR inhibitors in cancer therapy. J Hematol Oncol 2:45–61. 10.1186/1756-8722-2-45 19860903 10.1186/1756-8722-2-45 PMC2775749

[22] Jurca AA, Jurca AD, Petchesi CD et al (2025) Tuberous sclerosis complex: new insights into pathogenesis and therapeutic breakthroughs. Life 15:368–378. 10.3390/life15030368 40141713 10.3390/life15030368 PMC11944049

[23] Wagle N, Grabiner BC, Van Allen EM et al (2014) Response and acquired resistance to everolimus in anaplastic thyroid cancer. N Engl J Med 371:1426–1433. 10.1056/NEJMoa1403352 25295501 10.1056/NEJMoa1403352 PMC4564868

[24] Shah RB, Mehra R (2024) Renal cell carcinoma associated with TSC/MTOR genomic alterations: an update on its expanding spectrum and an approach to clinicopathologic work-up. Adv Anat Pathol 31:105–117. 10.1097/PAP.0000000000000419 37899532 10.1097/PAP.0000000000000419

[25] Yang P, Cornejo KM, Sadow PM et al (2014) Renal cell carcinoma in tuberous sclerosis complex. Am J Surg Pathol 38:895–909. 10.1097/PAS.0000000000000237 24832166 10.1097/PAS.0000000000000237 PMC4139167

[26] Guo J, Tretiakova MS, Troxell ML et al (2014) Tuberous sclerosis-associated renal cell carcinoma: a clinicopathologic study of 57 separate carcinomas in 18 patients. Am J Surg Pathol 38:1457–1467. 10.1097/PAS.0000000000000248 25093518 10.1097/PAS.0000000000000248

[27] Zhang H-Z, Xia Q-Y, Wang S-Y et al (2022) Low-grade oncocytic tumor of kidney harboring TSC/MTOR mutation: clinicopathologic, immunohistochemical and molecular characteristics support a distinct entity. Virchows Arch 480:999–1008. 10.1007/s00428-022-03283-x 35099634 10.1007/s00428-022-03283-x

[28] Farcaş M, Gatalica Z, Trpkov K et al (2022) Eosinophilic vacuolated tumor (EVT) of kidney demonstrates sporadic TSC/MTOR mutations: next-generation sequencing multi-institutional study of 19 cases. Mod Pathol 35:344–351. 10.1038/s41379-021-00923-6 34521993 10.1038/s41379-021-00923-6

[29] Sanguedolce F, Mazzucchelli R, Falagario UG et al (2024) Diagnostic biomarkers in renal cell tumors according to the latest WHO classification: a focus on selected new entities. Cancers (Basel) 16:1856. 10.3390/cancers16101856 38791935 10.3390/cancers16101856 PMC11120103

