# Supplementary figures and images for: Chromophobe thyroid carcinoma: a distinct entity associated with TSC gene alterations
Source: Virchows Arch. 2026 Jan 2;488(4):735–49. doi: 10.1007/s00428-025-04380-3 (PMC13053357; doi:10.1007/s00428-025-04380-3)

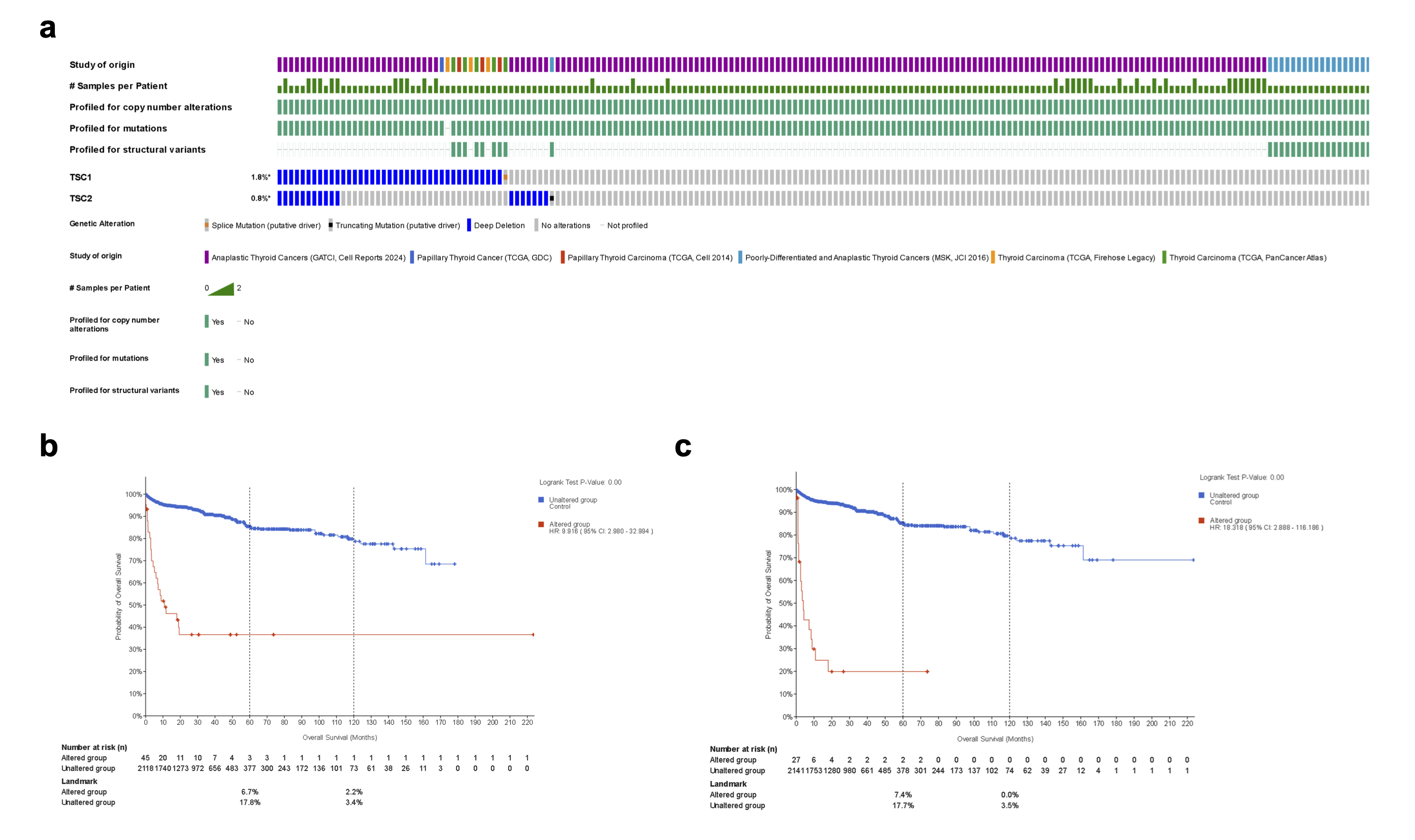

Supplement: Supplementary file 2 — High Resolution Image (17.5 MB) [file 428_2025_4380_MOESM1_ESM.tiff]
